# Nanofocusing of X-ray free-electron lasers by grazing-incidence reflective optics

**DOI:** 10.1107/S1600577515005093

**Published:** 2015-04-15

**Authors:** Kazuto Yamauchi, Makina Yabashi, Haruhiko Ohashi, Takahisa Koyama, Tetsuya Ishikawa

**Affiliations:** a Osaka University, 2-1 Yamada-oka, Suita, Osaka 565-0871, Japan; b RIKEN/SPring-8, 1-1-1 Kouto, Sayo-cho, Sayo-gun, Hyogo 678-5198, Japan; c JASRI/SPring-8, 1-1-1 Kouto, Sayo-cho, Sayo-gun, Hyogo 678-5198, Japan

**Keywords:** nanofocusing, X-ray free-electron laser, KB mirror, phase diagnosis

## Abstract

Total-reflection mirror devices for X-ray free-electron laser focusing are discussed in terms of optical design, mirror-fabrication technology, a wavefront diagnosis method and radiation-damage testing, as a review of the present status of the focusing optics at the SPring-8 angstrom compact free-electron laser (SACLA). Designed beam sizes of 1 µm and 50 nm, and spot sizes almost matching prediction have been achieved and used to explore topics at the forefront of natural science. The feasibility of these devices is determined to be sufficient for long-term and stable operation at SACLA by investigating the radiation-damage threshold and achievable accuracies in the mirror figure and alignment.

## Abstract 2

The current status and prospective applications of nanofocusing for X-ray free-electron lasers are presented.

## 1. Introduction   

X-ray free-electron laser (XFEL) sources have a peak brilliance 10^9^ greater than that of the most powerful third-generation synchrotron radiation sources and can emit high-intensity femtosecond pulses with full spatial coherence (Emma *et al.*, 2010 ▸; Ishikawa *et al.*, 2012 ▸). To enhance these characteristics, which would provide the opportunity to explore the forefront of natural sciences, focusing XFELs is a critical and urgent requirement. To accomplish this, there are a number of issues that must be addressed. First, high throughput is the most basic requirement for efficient usage of photons in achieving a higher fluence. Second, a sufficient spectral acceptance is necessary for making use of the full bandwidth (0.1%) of the XFEL. Third, distortion of the wavefront due to the optics themselves should be avoided wherever possible (Yamauchi *et al.*, 2005 ▸). Fourth, the optics should be aligned precisely to achieve optimal operation (Fukui *et al.*, 2013 ▸). To satisfy the first two conditions, total-reflection mirrors are the most favorable optics. However, it then becomes extremely difficult to satisfy the third and fourth items. The required precision in the figure of the mirror is below roughly a few nanometers (peak-to-valley) over the full spatial wavelength range to realise diffraction-limited operation (Yumoto *et al.*, 2006 ▸; Yamauchi *et al.*, 2002*a* ▸; Mimura *et al.*, 2007 ▸, 2008 ▸, 2010 ▸). To reduce speckle noise in the reflected beam, a much higher accuracy (less than 1 nm, peak-to-valley) is needed for spatial wavelengths of less than a few millimeters (Yamauchi *et al.*, 2005 ▸). Then, in the alignment of the mirrors, the grazing-incidence angle should be controlled to better than 0.5 × 10^−6^ rad (Matsuyama *et al.*, 2006 ▸).

In this paper we review the current achievements in focusing SACLA (SPring-8 angstrom compact free-electron laser) in terms of the optical configuration of the 1 µm and 50 nm focusing systems, required accuracies in optics, achievable accuracy in mirror fabrication, and a wavefront diagnosis method to monitor the mirror alignment and to realise long-term and stable operation.

## 2. Optical configuration of 1 µm and 50 nm focusing   

**Figure 1:**
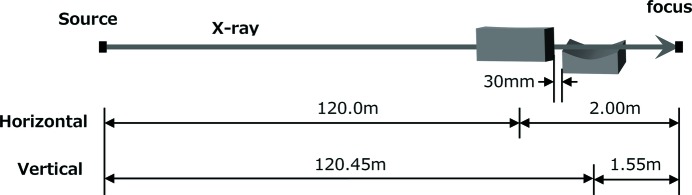
Optical configuration of the 1 µm focusing system of SACLA.

The optical configuration for 1 µm focusing of SACLA is shown in Fig. 1 ▸ (Yumoto *et al.*, 2012 ▸), and employs a Kirk­patrick–Baez (KB) geometry (Kirkpatrick & Baez, 1948 ▸). These design parameters are listed in Table 1 ▸. The respective distances from the end of the undulator to the centers of the upstream and downstream mirrors are 120 m and 120.45 m, and the respective focal lengths of the mirrors are 2.00 m and 1.55 m. To reduce the absorption and radiation damage on the mirror surfaces, they were coated with carbon and a sufficiently long substrate (420 mm) was employed. Here, the theoretical reflectivity is greater than 99%, even at the maximum incident angle of 1.63 mrad at the downstream edge of the mirror surface, and up to photon energies of 18 keV.

**Figure 2:**
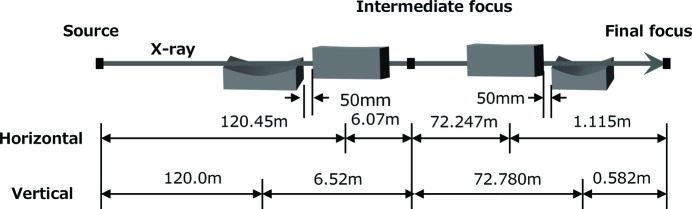
Optical configuration of the two-stage total-reflection 50 nm focusing system of SACLA.

To produce spot sizes as small as 50 nm, a shorter focal distance and larger aperture are needed to satisfy the geometrically required demagnification factor and large numerical aperture, respectively. However, a small focal distance imposes a serious problem when using intense XFEL: debris from the target irradiated by the XFEL pulses could severely degrade the optical performance of the mirror surface. Even when the mirror parameters satisfy the above requirements, an incident-beam size larger than the aperture of the mirror is necessary for producing the designed numerical aperture. However, this is difficult to achieve because of the small divergence of the XFEL beam, which is of the order of micro-radians, especially in a compact XFEL facility like SACLA (Ishikawa *et al.*, 2012 ▸). Even at hutch 5 the beam size is as small as 500 µm (FWHM). To overcome this difficulty, we developed a two-stage focusing system consisting of a pair of focusing mirrors in the KB geometry (Mimura *et al.*, 2014 ▸). Fig. 2 ▸ shows the configuration of this two-stage total-reflection focusing system developed at SACLA. For this configuration, we employed the first set of KB mirrors as pre-focusing optics to form a small source and effectively expand the beam size at the aperture of the second set of KB mirrors, which condense the X-rays as a final step. Total-reflection optics were employed here to enable the use of a wide range of wavelengths. The optical parameters are summarized in Table 2 ▸. The first KB mirrors were placed 120 m downstream from the end of the undulator to focus the XFEL pulses to a spot size of 3.6 µm × 3.2 µm with nearly 100% efficiency. The X-ray beam re-diverged after the intermediate focal point and propagated to the second set of KB mirrors located 72 m downstream from the first KB mirrors. The second set of KB mirrors has a larger aperture size (2.3 mm × 2.7 mm) due to the platinum coating that has large critical angle. Using this scheme, both a large numerical aperture and a long working distance were realised. The predicted wave-optic beam size, defined as the full width at half-maximum of the intensity profile at 9.9 keV, was 30 nm in the vertical and 55 nm in the horizontal directions, respectively, with a long working distance of 350 mm (Mimura *et al.*, 2014 ▸).

## 3. Accuracy of optics and fabrication technology   

The focusing performance of a coherent X-ray beam essentially depends on the wavefront aberration of the spherically condensing X-rays. The phase error φ induced by reflection on an imperfect mirror surface is given by
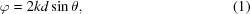
where θ and *k* are the grazing-incidence angle and the wavenumber of the X-ray, respectively, and *d* is the peak-to-valley height for the shape error of the mirror. To condense coherent X-rays into a spot size approaching the theoretical limit, all X-rays reflected on the mirror must interfere constructively at the focal point. The wavefront aberration reduces the degree of constructive interference at the focal point, and consequently distorts the focused-beam profile. To achieve diffraction-limited performance, Rayleigh’s criterion (Born & Wolf, 1999 ▸) requires that φ be less than π/2. To satisfy this criterion by employing equation (1), the required figure accuracy is estimated to be exceptionally high, namely 5 nm and 2 nm (peak-to-valley), respectively, for 1 µm and 50 nm focusing, in which X-ray energy was 10 keV (Yumoto *et al.*, 2012 ▸; Mimura *et al.*, 2014 ▸). In satisfying this accuracy in the spatial-wavelength range from a few tens of millimeters to the full length of the mirror, the side lobe near the focal point decreases significantly. Speckle noise in the reflected beam for the far field is much more sensitive to the figure error, especially in the short spatial-wavelength range. Sub-nanometer-height figure errors with a spatial-wavelength range from 0.1 mm to a few tens of millimeters give rise to problematic speckle noise (Yamauchi *et al.*, 2005 ▸).

**Figure 3:**
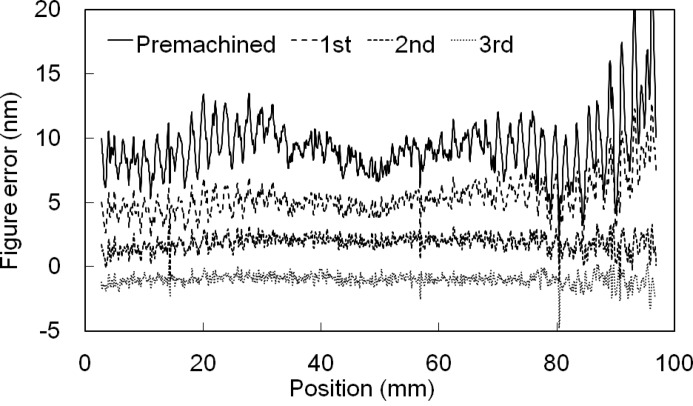
Typical deterministic figuring process with elastic emission machining and optical-stitching interferometry. Three trials of deterministic figuring reached 2 nm (peak-to-valley) for nearly every position on the mirror.

Generally, deterministic fabrication is utilized to produce highly accurate optics. This consists of figuring and figure-measurement methods. Currently, to satisfy accuracy requirements, ion-beam figuring (IBF) (Preda *et al.*, 2013 ▸; Schindler *et al.*, 2002 ▸), computer-controlled polishing (CCP) (Ando *et al.*, 1995 ▸; Aspden *et al.*, 1972 ▸) and elastic emission machining (EEM) (Mori *et al.*, 1987 ▸; Yamauchi *et al.*, 2002*b* ▸) are available as figuring methods. In our case, EEM was applied to fabricate the focusing optics for SACLA. This method involves chemical processing to etch the work surface using the surface reactivity of fine-powder particles. An atomically smooth surface is obtained in spatial wavelengths shorter than 50 µm by a global EEM planarization (Mori *et al.*, 2001 ▸). Then, a deterministic EEM process using nozzle heads eliminates figure errors in the spatial-wavelength range of greater than 50 µm up to the full length of the mirror surface. To measure the residual figure error for the deterministic figuring, we employed microstitching interferometry (MSI) (Yamauchi *et al.*, 2003 ▸) and relative-angle determinable-stitching interferometry (RADSI) (Mimura *et al.*, 2005 ▸), respectively, for short and long spatial-wavelength ranges in which lateral resolutions of RADSI and MSI are 300 µm and 20 µm. RADSI and MSI treat figure errors affecting the side lobe at the focal point and speckles in the far field, respectively. Thus, the mirror surfaces are mapped with respective height and spatial resolutions of 0.1 nm and 20 µm. The residual figure error typically obtained after deterministic figuring is shown in Fig. 3 ▸. This figure error is smaller than 2 nm (peak-to-valley), which is sufficiently small for satisfying Rayleigh’s criterion for 50 nm focusing of SACLA.

## 4. Wavefront diagnosis for mirror alignment   

**Figure 4:**
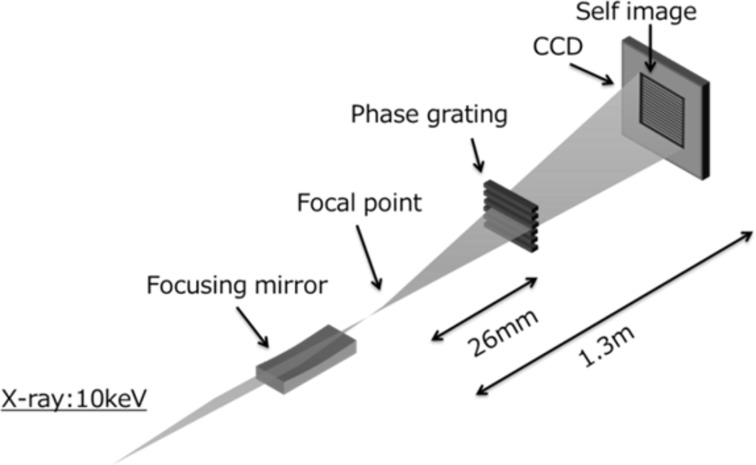
One-dimensional grating interferometry setup for estimating coma aberration.

**Figure 5:**
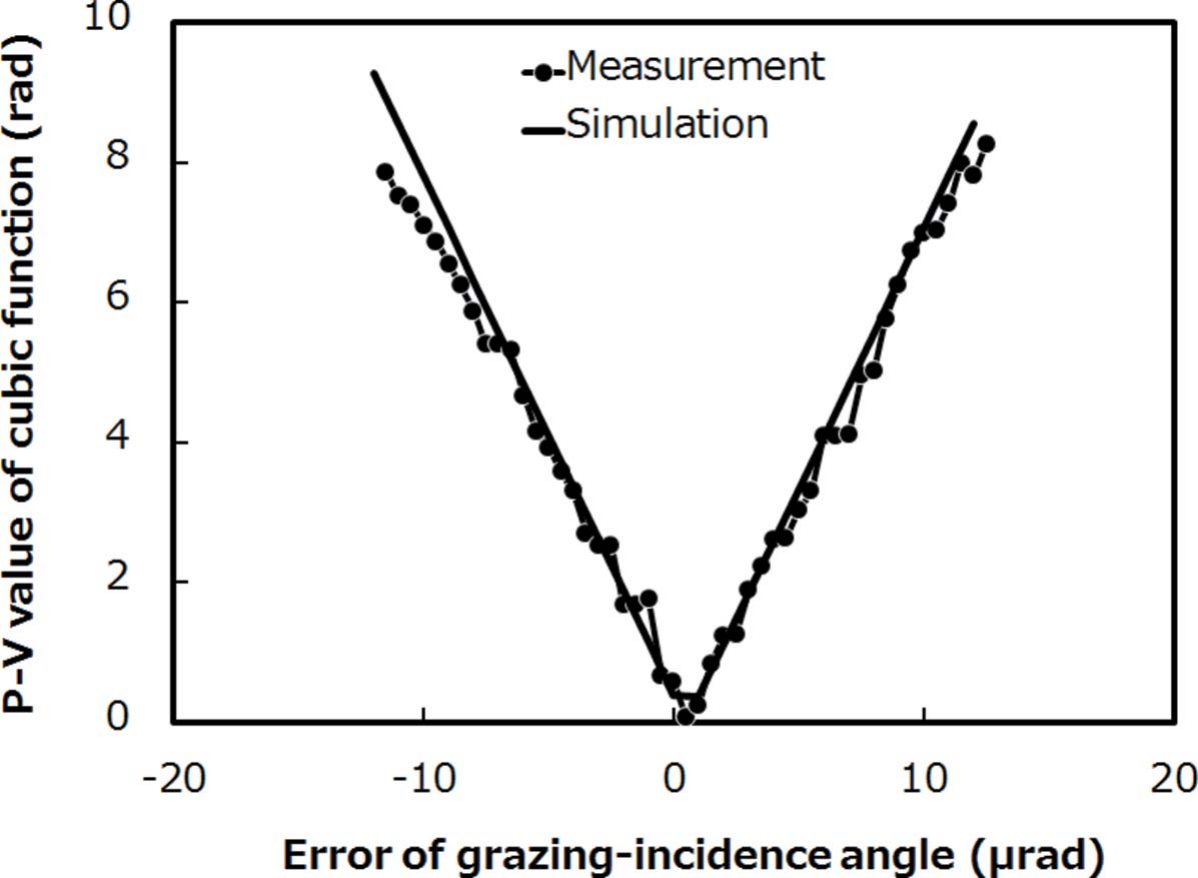
Typical relationship between grazing-incidence error and phase difference at the minimum and maximum of a cubic function extracted from the wavefront shape measured by grating interferometry. The solid curve shows the theoretically calculated relation.

To achieve the theoretically predicted focal-spot size, not only the figure accuracy of the mirror but also the alignment of the grazing-incidence angle is quite important, the error of which causes problematic coma aberration through the wavefront distortion with a cubic function shape. To satisfy Rayleigh’s criterion, the error in the grazing-incidence angle must not exceed 0.5 × 10^−6^ rad for 50 nm focusing of SACLA. Generally, alignment accuracy is evaluated by monitoring the beam profile using knife-edge scanning methods. In this way, the alignment is optimized by an iterative procedure of beam profiling and grazing-incidence-angle adjustment. This procedure is very time-consuming and frequently introduces a significant error in the beam profile from shape imperfections and/or vibrations of the scanner. Accordingly, the grazing-incidence-angle error often determines the achievable focal-spot size. In the evaluation of XFEL nanofocusing, a shot-by-shot method becomes essential in reducing the influence of the fluctuation of the focal position during beam profiling. To meet this requirement, we used single-grating interferometry (Takeda *et al.*, 2007 ▸; Matsuyama *et al.*, 2012 ▸; Weitkamp *et al.*, 2005 ▸; Diaz *et al.*, 2010 ▸; Wang *et al.*, 2011 ▸, 2013*a* ▸,*b* ▸; Rutishauser *et al.*, 2011 ▸, 2012 ▸; Yuan *et al.*, 2011 ▸; Berujon & Ziegler, 2012 ▸; Merthe *et al.*, 2013*a* ▸,*b* ▸; Yamauchi *et al.*, 2005 ▸) based on the Talbot effect (Talbot, 1836 ▸). Thus, we tested the sensing capability of the coma aberration generated by the grazing-incidence-angle error of a mirror for 50 nm focusing of SACLA. The setup for this is shown in Fig. 4 ▸; the energy of the X-rays was 10 keV. A tantalum phase grating (2.5 mm pitch; NTT Advanced Technology Corporation) fabricated on a thin SiC membrane with a thickness of less than 1 µm was used. A grating with a thickness of 1.4 mm was designed to behave as a π/2-phase shifter for 10 keV X-rays. The grating was placed 26 mm downstream from the focal point. The formed self-image was recorded by a CCD camera (AA40MOD and ORCA-R2, Hamamatsu Photonics) placed 1.3 m downstream from the focal point. This configuration yields a 50× magnification of the grating image. The effective pixel size and field of view of the camera were 6.5 µm and 8.736 mm × 6.656 mm, respectively. The pulse energy employed was 10 µJ with 1/40 attenuation by a silicon crystal with an appropriate thickness. Self-images were obtained by single-shot irradiation. Coma aberration correlates linearly with the amplitude of the cubic function in the wavefront error. To accurately evaluate the coma aberration induced by the grazing-incidence-angle error, best-fit quadratic functions were removed from the reconstructed wavefront shape. The obtained wavefront shape then appeared as a cubic function with no significant higher-order polynomials, implying that the figure accuracy of the mirror was sufficiently high. The amplitudes of the higher-order polynomials were less than λ/10 in this case. We measured the phase difference between the minima and maxima of the cubic function by changing the grazing-incidence angle (pitched at 0.5 × 10^−6^ rad) from −12 × 10^−6^ rad to 12 × 10^−6^ rad at the optimally aligned angle. In addition, we calculated the phase difference at the angles employed in the experiment. For this calculation, the mirror was assumed to have an ideal shape. Fig. 5 ▸ plots the experimental and calculated results, which are in good agreement. The phase difference at the minimum, in which the grazing-incidence angle is optimal, was small enough to satisfy Rayleigh’s criterion. Single-grating interferometry is an appropriate method for evaluating the aberration shot-by-shot. We are planning to install such a system in the 50 nm focusing optics of SACLA to drastically simplify the alignment procedure and shorten the alignment time.

## 5. Focusing performance and discussion   

**Figure 6:**
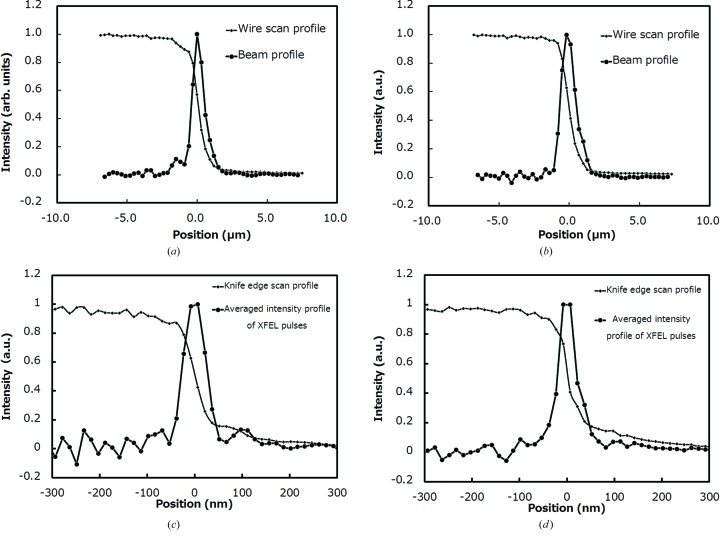
Focused-beam profiles in the horizontal direction (*a*) and vertical direction (*b*) for 1 µm focusing and in the horizontal direction (*c*) and vertical direction (*d*) for 50 nm focusing of SACLA.

**Figure 7:**
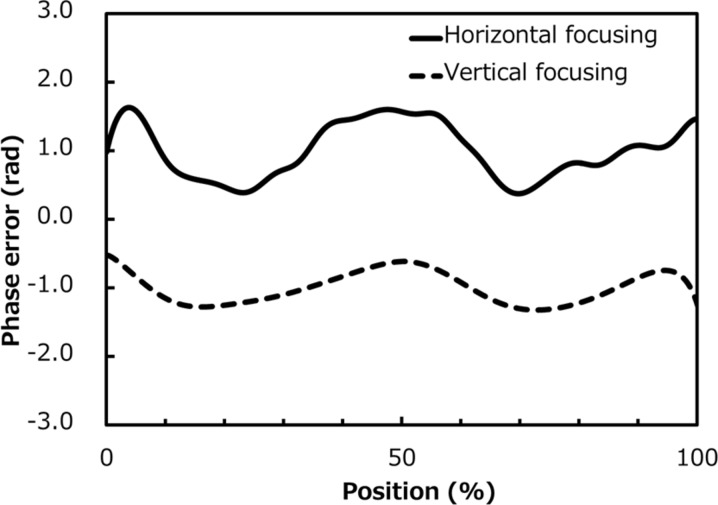
Wavefront aberration due to imperfections in the downstream KB mirrors measured by grating interferometry. Quadratic and cubic shapes are removed. Upper and lower curves are for horizontal and vertical focusing mirrors, respectively. Position is normalized by the full-aperture size of each mirror.

Optical systems for 1 µm and 50 nm focusing were installed in BL3 of SACLA. Wire scan and/or knife-edge scan methods were applied to roughly investigate the focused-beam profiles. These methods are capable of evaluating 1 µm focusing but are not suitable for 50 nm focusing, in which the beam profile is estimated to be significantly larger because of the fluctuation of the focal point. Measured results are shown in Figs. 6(*a*)–6(*d*) ▸. With the 1 µm focusing optics, the beam size defined by full width at half-maximum was 0.95 µm × 1.20 µm in the horizontal and vertical directions, respectively (at a photon energy of 10 keV with an almost ideal reflectivity of 97%). The flux density of the focused beam was enhanced to be 40000 times greater than that of the unfocused one. A peak power density at a maximal pulse energy of 0.4 mJ was estimated to be 5 × 10^18^ W cm^−2^ when assuming a pulse duration of 10 fs (Emma *et al.*, 2010 ▸; Inubushi *et al.*, 2012 ▸). With the 50 nm focusing optics, the beam size was 45 nm × 55 nm in the vertical and horizontal directions, respectively, at a photon energy of 9.9 keV, which was slightly larger than the predicted 30 nm in the vertical direction. As mentioned above, the knife-edge-scan method is not suitable for evaluating the 50 nm focusing optics. Grating interferometry, using a shot-by-shot method, was employed to understand the exact wavefront aberration. In this case, the cubic function corresponding to the coma aberration was reduced to a negligible value by precisely aligning the grazing-incidence angle, and higher-order polynomial elements were extracted to investigate the wavefront error due to the imperfections of the mirror figure. Results of this test are shown in Fig. 7 ▸. The impact of the mirror imperfection is less than 1 rad both in the horizontal and vertical directions, which satisfies Rayleigh’s criterion by a comfortable margin. In addition, we found that the phase error here is almost the same as that estimated by the residual figure error on the downstream KB mirrors. In the two-stage optics for 50 nm focusing, grazing-incidence angles of the downstream mirrors are about three times larger than those of the upstream mirrors, which means that the figure errors of the downstream mirrors affect the wavefront error with a threefold increase in sensitivity compared with those of the upstream mirrors.

Accordingly, we concluded that the wavefront error originates in the imperfections of the downstream mirrors. Meanwhile, the wavefront error is small enough for performing diffraction-limited focusing, and the beam size is theoretically expected to be 30 nm × 55 nm. By these considerations, with an estimated pulse duration of 10 fs, peak power density is expected to be nearly 10^20^ W cm^−2^.

**Figure 8:**
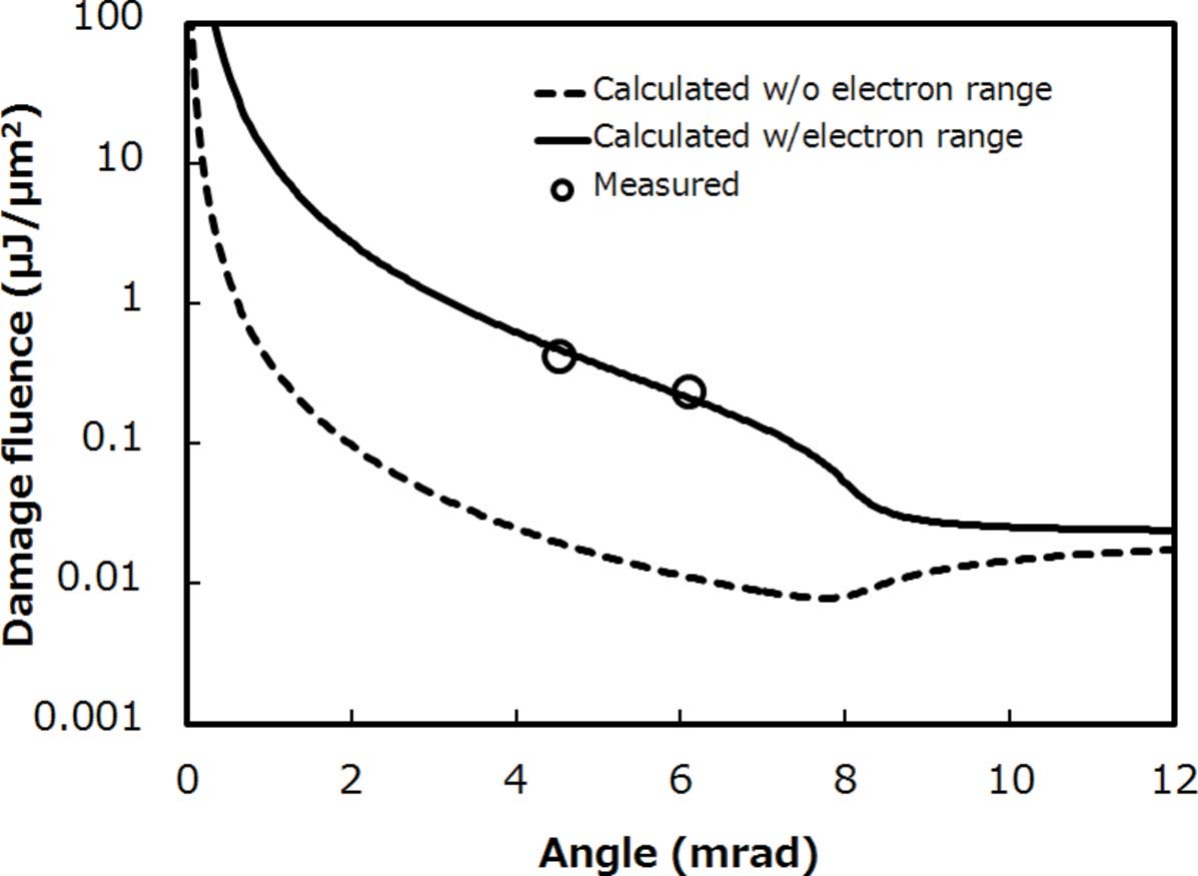
Relationship between grazing-incidence angle and damage threshold. Solid and dashed curves show thresholds with and without consideration of electron-collision length. Circles are measurements using the 1 µm-focused configuration at SACLA.

Finally, we discuss the radiation-damage threshold in the grazing-incidence mirror under the conditions employed at SACLA. The damage-threshold fluence of platinum film under normal incidence was reported to be 0.023 µJ µm^−2^ by an irradiation test using a 1 µm-focused beam; this reflects a single-atom dose of 0.52 eV atom^−1^ (Koyama *et al.*, 2013*a* ▸). In the grazing-incidence case, damage fluence can be written as
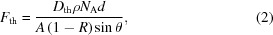
where ρ, *N*_A_, *A*, *R* and θ are the density, Avogadro’s constant, atomic weight, reflectivity and grazing-incidence angle, respectively. *D*_th_ is an energy dose per atom at aberration threshold and assumed here to be the melting dose. The variable *d* is the energy deposition depth, given by *d* = 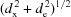, where *d*_x_ is the X-ray penetration depth calculated with the absorption coefficient μ_g_(θ) as
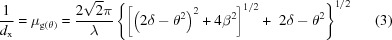
where δ and β are related to the complex index* n* = 1 − δ + *i*β, and λ and θ are the X-ray wavelength and grazing-incidence angle, respectively. The variable *d*_e_ is the electron collision length (Koyama *et al.*, 2013*a* ▸,*b* ▸) and is used here as a fitting parameter. Fig. 8 ▸ shows the damage-threshold fluence as a function of grazing-incidence angle. Solid and dashed lines indicate a *d*_e_ of zero and 35 nm, respectively. The fluence under the large grazing-incidence angle asymptotically approaches 0.023 µJ µm^−2^, which occurs under normal-incidence conditions. The two circles reflect measured damage-threshold fluences defined as maximum survivable fluence after a 10^4^ shot irradiation. In this experiment the 1 µm-focused beam of SACLA was used with attenuators to supply the required fluence. As shown in Fig. 8 ▸, the experimentally obtained damage threshold near the critical angle was larger than 0.2 µJ µm^−2^ and agrees with that estimated using an electron-collision length of 35 nm. Such short collision length was neglected in the case of normal incidence, but has a significant role in reducing radiation damage by a factor of ten under grazing-incidence conditions. In the focusing optics at SACLA, we can estimate a maximum fluence of 0.01 µJ µm^−2^ at the downstream KB mirrors under a pulse energy of 0.5 mJ. This implies that the actual operation conditions have a sufficient margin for the ablation threshold and clearly shows the feasibility of the mirror optics for long-term and stable operation at XFEL.

To achieve further smaller spot size, another two-stage focusing system with multilayer mirrors is now under development. The radiation damage threshold of the multilayer has already been characterized to survive under the XFEL irradiation (Kim *et al.*, 2015 ▸). The expected beam size is roughly 5–6 nm square, and the peak power will reach more than 10^21^ W cm^−2^.

## Figures and Tables

**Table 1:** Parameters of the 1 µm focusing KB optics

	Horizontal focusing mirror	Vertical focusing mirror
Mirror shape	Elliptical cylinder	Elliptical cylinder
Substrate material	Quartz	Quartz
Coating	Carbon	Carbon
Mirror substrate size	420 × 50 × 50 mm	420 × 50 × 50 mm
Grazing angle	1.50 mrad	1.55 mrad
Focal length	1.55 m	2.00 m
Semi-major axis	51 m	51 m
Semi-minor axis	18.7 mm	21.9 mm

**Table 2:** Parameters of the 50 nm focusing two-stage KB optics

	Upstream KB mirrors		Downstream KB mirrors	
	Horizontal focusing mirror	Vertical focusing mirror	Horizontal focusing mirror	Vertical focusing mirror
Mirror shape	Elliptical cylinder	Elliptical cylinder	Elliptical cylinder	Elliptical cylinder
Substrate material	Quartz	Quartz	Quartz	Quartz
Coating	None	None	Platinum	Platinum
Substrate size	400 × 50 × 50 mm	400 × 50 × 50 mm	500 × 50 × 50 mm	465 × 50 × 50 mm
Grazing angle	1.5 mrad	1.5 mrad	5.5 mrad	5.0 mrad
Focal length	6.070 m	5.520 m	1.115 m	0.582 m
Distance from source	120.45 m	120 m	72.247 m	72.780 m
Semi-major axis	63.26 m	63.26 m	36.68 m	36.68 m
Semi-minor axis	40.6 m	42.0 m	49.4 m	32.6 m
